# *Salmonella* Breaks Tumor Immune Tolerance by Downregulating Tumor Programmed Death-Ligand 1 Expression

**DOI:** 10.3390/cancers12010057

**Published:** 2019-12-24

**Authors:** Man-Chin Chen, Christian Ronquillo Pangilinan, Che-Hsin Lee

**Affiliations:** 1 Department of Biological Sciences, National Sun Yat-sen University, Kaohsiung 80424, Taiwan; menny628@hotmail.com (M.-C.C.); chtianbiol@gmail.com (C.R.P.); 2 Department of Medical Research, China Medical University Hospital, China Medical University, Taichung 40402, Taiwan; 3 Department of Medical Laboratory Science and Biotechnology, Kaohsiung Medical University, Kaohsiung 80708, Taiwan; 4 Doctoral Degree Program in Marine Biotechnology, National Sun Yat-sen University, Kaohsiung 80424, Taiwan

**Keywords:** *Salmonella*, immune checkpoint, programmed death-ligand 1, T cell

## Abstract

Immunotherapy is becoming a popular treatment modality in combat against cancer, one of the world’s leading health problems. While tumor cells influence host immunity via expressing immune inhibitory signaling proteins, some bacteria possess immunomodulatory activities that counter the symptoms of tumors. The accumulation of *Salmonella* in tumor sites influences tumor protein expression, resulting in T cell infiltration. However, the molecular mechanism by which Salmonella activates T cells remains elusive. Many tumors have been reported to have high expressions of programmed death-ligand 1 (PD-L1), which is an important immune checkpoint molecule involved in tumor immune escape. In this study, Salmonella reduced the expression of PD-L1 in tumor cells. The expression levels of phospho-protein kinase B (P-AKT), phospho-mammalian targets of rapamycin (P-mTOR), and the phospho-p70 ribosomal s6 kinase (P-p70s6K) pathway were revealed to be involved in the Salmonella-mediated downregulation of PD-L1. In a tumor-T cell coculture system, Salmonella increased T cell number and reduced T cell apoptosis. Systemic administration of Salmonella reduced the expressions of PD-L-1 in tumor-bearing mice. In addition, tumor growth was significantly inhibited along with an enhanced T cell infiltration following Salmonella treatment. These findings suggest that Salmonella acts upon the immune checkpoint, primarily PD-L1, to incapacitate protumor effects and thereby inhibit tumor growth.

## 1. Introduction

Conventional therapies, such as surgery, radiation therapy, and chemotherapy, have been used to destroy tumor cells directly, and immunotherapy has provided another option for patients who are suffering from the inefficiency of conventional treatments, primarily due to the emergence of high-grade malignant phenotypes and tumor cell drug resistance. In using immunotherapy for cancer treatment, the co-stimulatory and co-inhibitory immune checkpoints play an important role in naïve T cell biology. Cluster of differentiation 28 (CD28) was the first molecule recognized as a co-stimulatory checkpoint. The engagement of T cell receptor (TCR) with CD28 induces naïve T cell proliferation and differentiation. Conversely, the co-inhibitory molecules, such as cytotoxic T lymphocyte antigen 4 (CTLA-4), programmed cell death protein 1 (PD-1), and lymphocyte-activation gene 3 (LAG-3), among others regulate T cell cytokine secretion (e.g., interferon-γ, IFN-γ), proliferation and apoptosis [1]. In addition to that, PD-L1—one of the ligands of PD-1—was thought to inactivate T cell proliferation, cytokine production, and cytotoxicity. Interestingly, high levels of immunosuppressive PD-L1 were found on the surface of human metastatic melanoma, lung, and breast cancers. Patients who failed to respond to anti-PD-1 antibodies showed higher circulating exosomal PD-L1 expression [2]. Recently, monoclonal antibodies targeting CTLA-4 (Ipilimumab and Tremelimumab), PD-1 (Nivolumab, Pembrolizumab, and Pidilizumab), and PD-L1 (Avelumab, Atezolizumab, and Durvalumab) have already acquired US Food and Drug Administration (FDA) approval for the treatment of highly metastatic melanoma and metastatic non-small cell lung carcinoma, while others are still undergoing clinical and pre-clinical trials [3]. Despite the progress in tumor immunotherapy using checkpoint inhibitors, some drawbacks have been observed including the relatively low response due to the development of resistance to monoclonal antibodies in some patients and the occurrence of autoimmune disease. In addition to that, the price of checkpoint inhibitors is too high to provide complete treatment for patients. These reasons have led us to investigate further into a more effective strategy for tumor therapy.

The use of bacteria in cancer therapy can be traced back to the 19th century [4]. Complex tissue organization and irregular blood vessel distribution caused by tumor hypoxia present difficulties in delivering antitumor agents. Interestingly, this kind of hypoxia restriction in tumor tissue allows colonization by anaerobic bacteria [5]. Upon localizing *Salmonella* to the tumor site, an immune response starts from innate immunity, including neutrophils, natural killer cells (NK), macrophages, and dendritic cells (DC), against the pathogen immediately. Subsequently, antigen-presenting cells (APCs) activate the adaptive response, including B and T cells, to eradicate the specific pathogens, eventually providing long-lasting immunity and memory [6,7,8]. In one study, *Bifidobacterium* combined with anti-PD-L1 antibodies induced CD8^+^ T cell accumulation and achieved the effect of slowing down melanoma tumor growth [9]. In addition to these common co-inhibitory agents, Zhao et al. indicated that using Pimozide, a psychiatric drug and dopamine antagonist, combined with attenuated *Salmonella* carrying PD-1-specific siRNA, could be a potential strategy in recruiting infiltrating CD4^+^ and CD8^+^ T cells, thus enhancing the anti-melanoma effect [10].

So far, the Gram-negative, facultatively anaerobic *Salmonella* being used in cancer therapy has been well documented and widely studied. *Salmonella-*mediated tumor therapy has many advantages including targeting tumor hypoxia, mobility, therapeutic genes, or protein delivery, enhancing chemosensitivity at low cost [11]. The lipopolysaccharide (LPS) of *Salmonella* enhances cytokine production and antitumor activities via Toll-like receptor 4 (TLR4) signaling [12]. *Salmonella* downregulated the expression of indoleamine 2, 3-dioxygenase 1 (IDO), another immune checkpoint, leading to a reduced kynurenine synthesis and increased CD8^+^ T cell infiltration [13]. Furthermore, the use of T-cell-deficient mice allowed us to find that T cells participate in antitumor activities of *Salmonella* [8]. Previous observations provided hints that *Salmonella* plays an important regulatory role in the immune system to reduce tumor growth. Herein, we unveiled the mechanism of how *Salmonella* affects the immune system through the regulation of PD-L1—an immune checkpoint molecule necessary for cancer cells to evade antitumor immune responses.

## 2. Results

### 2.1. PD-L1 Was Downregulated by Salmonella

**Figure 1:**
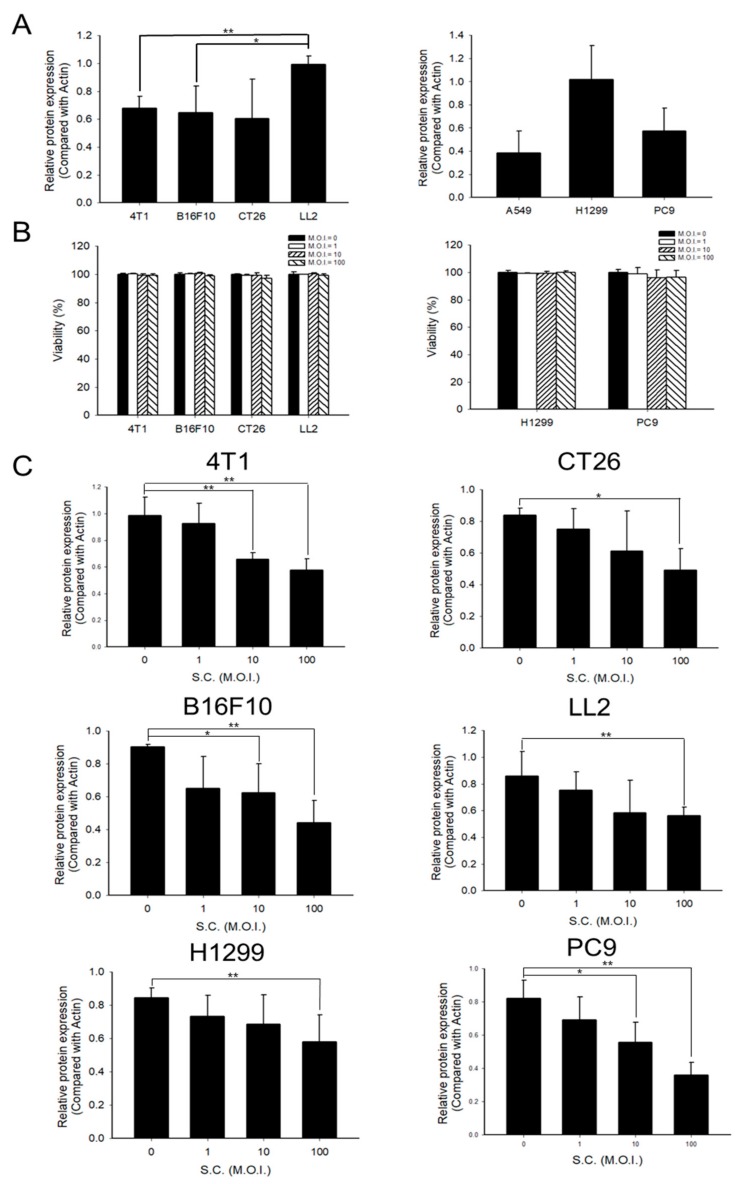
The expression levels of programmed death-ligand 1 (PD-L1) in various cancer cell lines and the effects of *Salmonella* (S.C.) treatment. (**A**) Quantified band intensities of respective PD-L1 proteins in various cancer cell lines. (**B**) Cell viability of tumor cells infected with *Salmonella* (multiplicity of infection (M.O.I.) = 0, 1, 10, and 100). (**C**) *Salmonella* dose-dependently inhibited PD-L1 expression. Tumor cells were seeded (Seeding density: 4T1 cells 5 × 10^5^, CT26 and B16F10 cells 3 × 10^5^, H1299 5 × 10^5^, LL2, and PC9 cells 7 × 10^5^) in 6-well plates. Each well was treated with respective doses (M.O.I. = 0, 1, 10, and 100) of *Salmonella* for 90 min and cultured in free Dulbecco’s Modified Eagle Medium (DMEM )with 1% Gentamicin for 24 h; cells were then lysed and tested for PD-L1 expression by Western blotting (please see Supplementary Figure S1). Statistical significance was calculated using Student’s *t*-test. *, *p* < 0.05; **, *p* < 0.01.

PD-L1 is overexpressed on the surface of tumor cells [14]. First, the protein levels of PD-L1 were measured in murine cancer cell lines and human non-small cell lung cancer cell lines (NSCLC). LL2 and H1299 showed higher PD-L1 expression than other murine and NSCLC (Figure 1A and Figure S1A). As *Salmonella* can influence the protein expression of tumor cells, we treated the tumor cell lines with different multiplicity of infection (M.O.I.) of *Salmonella*. To test the tolerance of tumor cells to *Salmonella*, murine cell lines and NSCLC were infected with 0, 1, 10, and 100 M.O.I. *Salmonella* for 1.5 hours and then the cell viability was recorded. Data (Figure 1B) revealed that even at the highest dose (M.O.I. = 100), *Salmonella* did not significantly affect cell viability. Similar results were demonstrated by Ingram et al. using viable and up to 25 M.O.I. *Salmonella* against murine embryonic fibroblasts (MEFs), while combined exposure with IFN-γ would cause cell death [15]. Figure 1C and Figure S1B showed that *Salmonella* decreased the expression of PD-L1 dose-dependently both in murine and human cancer cell lines. *Salmonella* regulated protein expression, including PD-L1, within tumor cells. Another immune checkpoint, PD-L2, which is another ligand for PD-1, did not elicit similar results. 

### 2.2. Phosph-Protein Kinase B (P-AKT)/Phosph-Mammalian Targets of Rapamycin (P-mTOR) Pathway Was Involved in Regulating PD-L1 Expression after Salmonella Treatment

**Figure 2:**
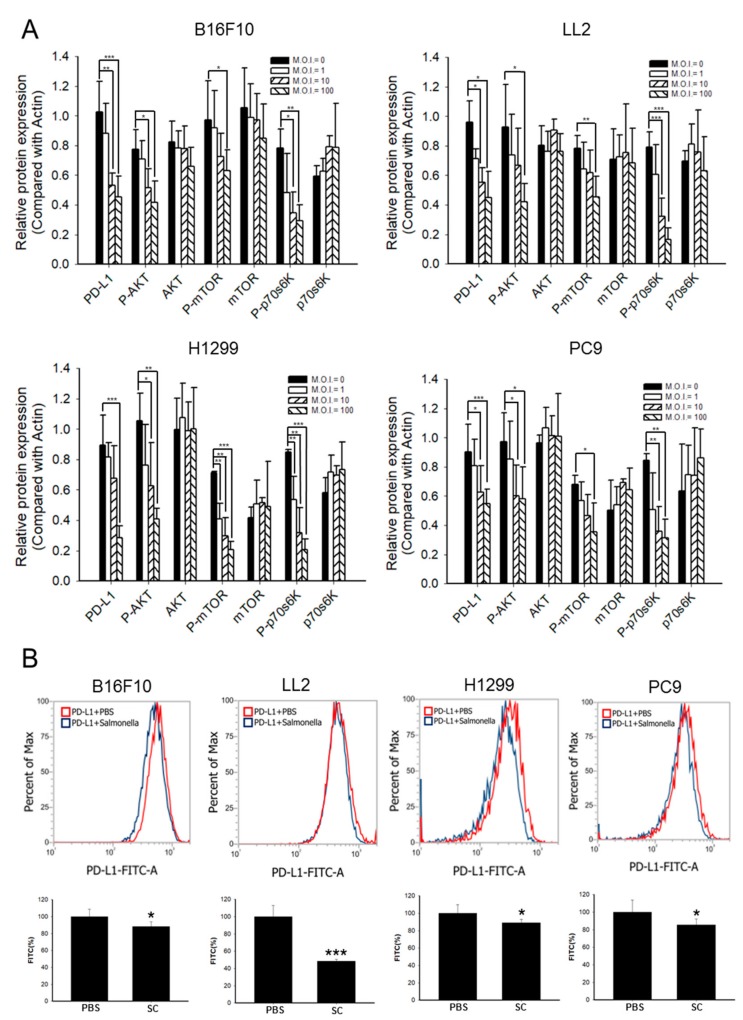
*Salmonella* (S.C.) reduced the expression of programmed death-ligand 1 (PD-L1. Cancer cells were seeded in 6-well plates. Afterwards, cells were treated with different doses (multiplicity of infection (M.O.I.) = 0, 1, 10, and 100) of *Salmonella* for 90 min and cultured in free Dulbecco’s Modified Eagle Medium (DMEM) with 1% Gentamicin for 24 h. (**A**) Quantified band intensities of respective proteins in *Salmonella*-infected cell lines. B16F10, LL2, H1299, and PC9 cells were lysed and their PD-L1 and protein kinase B (AKT)/mammalian targets of rapamycin (mTOR)/ p70 ribosomal s6 kinase (p70s6K) markers were examined by Western blotting (please see Supplementary Figure S2). (**B**) Cells were treated with 100 M.O.I. *Salmonella* for 90 min and PD-L1 expression analysis on tumor cells by flow cytometry was performed after fixing cells in 70% ethanol at −30 °C overnight; the bar graph also shows the quantified value of PD-L1-FITC-A. *, *p* < 0.05; **, *p* < 0.01; ***, *p* < 0.001.

Previous studies showed that PD-L1 was regulated by the phosphoinositide-3-kinase–protein kinase B (PI3K)/protein kinase B (AKT)/mammalian targets of rapamycin (mTOR) pathway, which promoted cancer protein synthesis, survival, proliferation, and apoptosis [16]. We suggested that the AKT/mTOR axis might be involved in the effect of *Salmonella* on PD-L1 expression. As shown in Figure 2A and Figure S2A, *Salmonella* could downregulate PD-L1 expression in a dose-dependent manner. Simultaneously, the decreased expression of phosphor-AKT, phosphor-mTOR, and phosphor-p70s6K in B16F10, LL2, H1299, and PC9 cell lines was observed after *Salmonella* treatment. Moreover, the expression of PD-L1 was significantly reduced on the cell surface after the murine and human NSCLC cell lines were treated with 100 M.O.I. *Salmonella,* as demonstrated by flow cytometry analysis (Figure 2B and Figure S2B). These findings suggested that reduction of PD-L1 expression in tumor cells was correlated with inhibition of the AKT/mTOR/p70s6K signaling pathway after *Salmonella* treatment. Based on these data, we utilized 100 M.O.I. of *Salmonella* in the succeeding experimental models as we found that *Salmonella* will not affect cell viability even with significantly decreased PD-L1 through the AKT/mTOR pathway.

### 2.3. Salmonella-Treated Cells Influenced the Cell Cycle, Apoptosis, and Function of Immune Cells 

**Figure 3:**
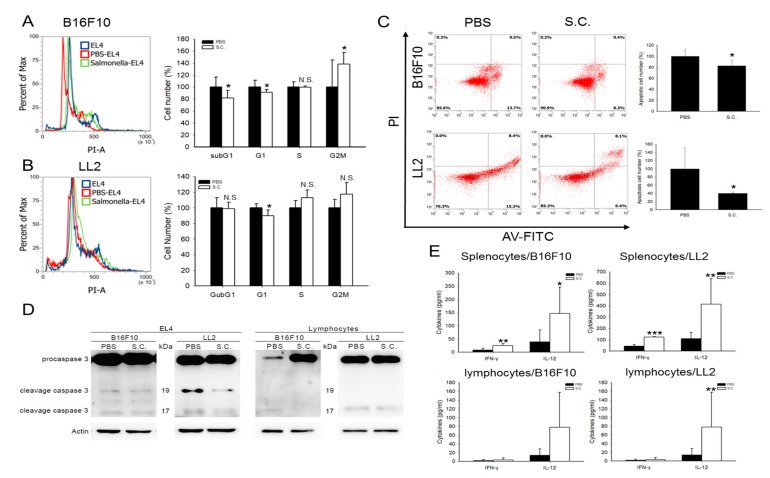
*Salmonella* (S.C.) affected cell cycle and apoptosis in T cells. *Salmonella* induced G1 block in the EL4 cell cycle when co-cultured with (**A**) B16F10 and (**B**) LL2 that have been subjected to *Salmonella* treatment at 100 multiplicity of infection (M.O.I.); cells were stained with propidium iodine, and the cell cycle analysis was performed by flow cytometry. (**C**) EL4 cells were stained with Annexin V and the frequency of apoptotic cells was measured after being co-cultured with *Salmonella*-infected B16F10 and LL2 cells. (**D**) EL4 cells and lymphocytes were co-cultured with B16F10 and LL2 cells prior to harvesting. Caspase 3 was analyzed by Western blotting. (**E**) *Salmonella* induced Interferon-γ (IFN-γ) and interleukin-12 (IL-12) cytokine secretion in splenocytes and lymphocytes which were co-cultured with *Salmonella*-infected B16F10 and LL2 cells. *, *p* < 0.05; **, *p* < 0.01; N.S., not significant. Compared with phosphate buffered saline (PBS).

PD-L1 binds to its receptor PD-1 to “turn off” the function of immune cells [17]. To mimic the interaction of a T cell encounter with tumor PD-L1, we chose the EL4 cell line that is rich in PD-1 expression, for the functional assay [18]. Flow cytometry analysis revealed that the cell cycle of EL4 at subG1 and G1 phase was significantly reduced and G2M was increased after co-culturing with *Salmonella*-treated B16F10 cells (Figure 3A). However, only the G1 phase was significantly decreased in the LL2 cell line (Figure 3B). Furthermore, we found that the number of EL4 apoptotic cells were decreased both in co-cultured *Salmonella*-treated B16F10 and LL2 cell lines (Figure 3C). Figure 3D and Figure S3 showed that cells treated with *Salmonella* decreased the cleavage caspase 3 (17/19 kDa) expression of co-cultured EL4 and lymphocytes. These results suggested that the *Salmonella*-treated tumor cells lost the inhibition of T cell proliferation function, such as activated caspase 3; the activated form of caspase 3 triggers a caspase cascade that eventually leads to cell death by apoptosis [19,20]. These results prompted us to further investigate the interaction between T cells and tumor cells as affected by *Salmonella* treatment. Since the spleen is known to be enriched with naïve lymphocytes, we co-cultured mice splenocytes with tumor cells after *Salmonella* treatment for 1.5 h. Figure 3E indicated that Interferon-γ (IFN-γ) and interleukin-12 (IL-12) were significantly increased in splenocytes that have been co-cultured with *Salmonella*-treated B16F10 and LL2. These evidences unveiled that *Salmonella* affects the cell cycle, apoptosis, and function of immune cells, T cells in particular, by reducing the expression of PD-L1 in tumor cells.

### 2.4. Salmonella Downregulated PD-L1 and Caspase 3 through AKT Signal Pathway in Tumor and EL4 Cells, Respectively

**Figure 4:**
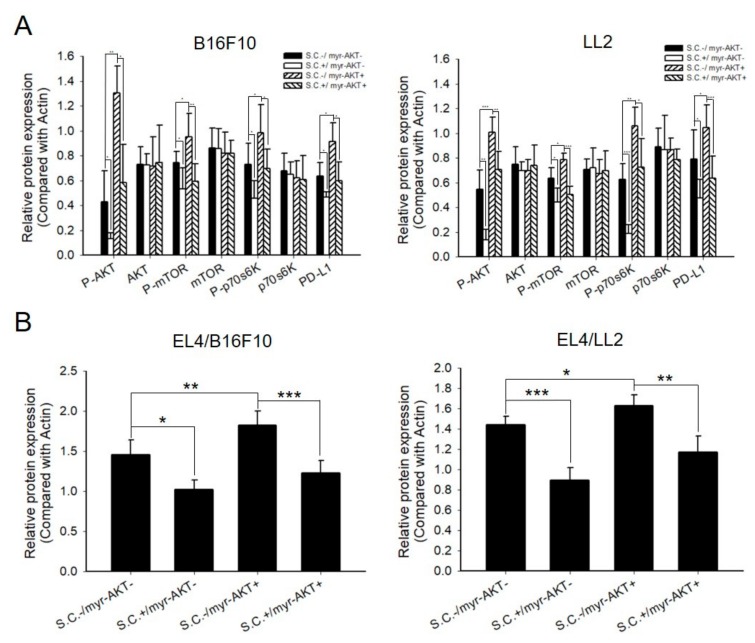
Constitutively active protein kinase B (AKT) abolished the inhibition of programmed death-ligand 1 (PD-L1) and caspase 3 expression which were reversed by *Salmonella* (S.C.). Tumor cells were seeded in 6-well plates and transfected with constitutively active protein kinase B (AKT) for 16 hours and then treated with *Salmonella* 100 multiplicity of infection (M.O.I.) for 90 min. Subsequently, cells were cultured in free DMEM with 1% Gentamicin for 24 h. (**A**) Quantified band intensities of respective proteins of B16F10 and LL2 cells were lysed and the protein kinase B (AKT)/mammalian targets of rapamycin (mTOR)/ p70 ribosomal s6 kinase (p70s6K) and PD-L1 markers were analyzed. (**B**) Quantified band intensities of respective proteins of EL4 cells were co-cultured with *Salmonella*-treated tumor cells for 24 h, and the caspase 3 (17/19 kDa) expression was analyzed. (For Western blot analysis, please see Supplementary Figure S4) The inserted values indicate protein expression compared to β-actin. Mean ± Standard Deviation (SD) represented independent expression. Statistical significance was calculated using Student’s *t*-test. *, *p* < 0.05; **, *p* < 0.01; ***, *p* < 0.001.

To validate the involvement of the AKT signaling pathway in tumor and immune cells, we delivered constitutively active AKT into tumor cells and then treated them with *Salmonella*. Tumor cells were collected to examine the PD-L1 expression and signaling pathway by Western blotting. Figure 4A and Figure S4A showed that while the expression of PD-L1 was significantly increased in B16F10 and LL2 cell lines having constitutive expression of AKT alongside the increased phosphorylation of AKT/mTOR/p70s6k, *Salmonella* treatment countered these effects. In addition, the expression of cleaved caspase 3 in EL4 cells, which were co-cultured with tumor cells, was decreased (Figure 4B and Figure S4B). These results indicated that the AKT/mTOR signaling pathway played an essential role in *Salmonella*-regulated PD-L1 expression.

### 2.5. Salmonella Enhanced T Cell Infiltration and Suppresses Tumor Growth via Downregulation of PD-L1 In Vivo

**Figure 5:**
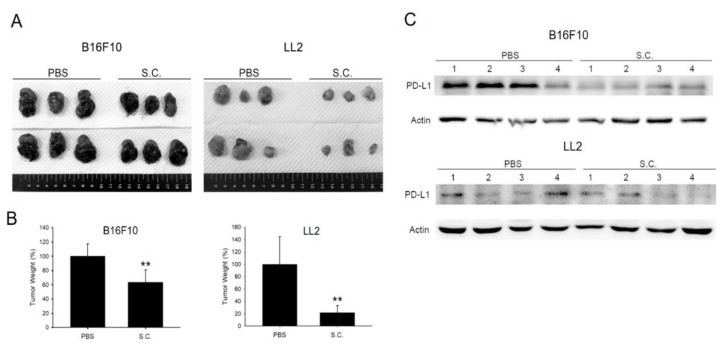
*Salmonella* (S.C.) inhibited tumor growth in vivo and enhanced T cells infiltration by downregulating programmed death-ligand 1 (PD-L1). (**A**) Tumor tissue size was observed and (**B**) measured on day 15 and 21 for B16F10 (*n* = 6) and LL2 (*n* = 6), respectively. **, *p* < 0.01. (**C**) PD-L1 expression of B16F10 and LL2 tumors were analyzed by Western blotting. Each group was repeated four times.

**Figure 6:**
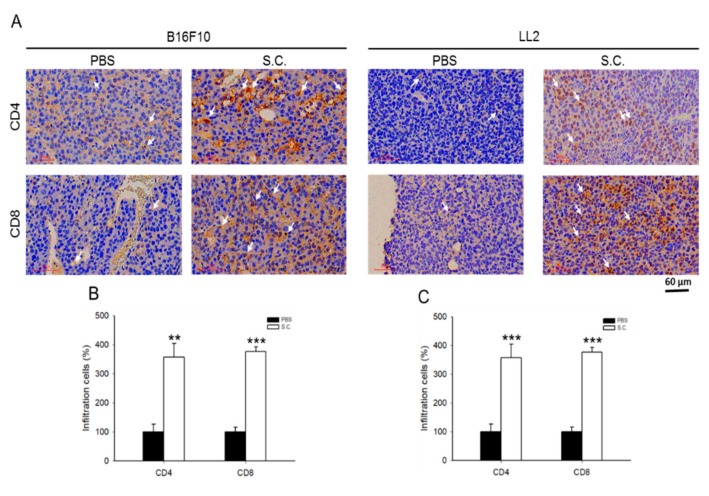
*Salmonella* (S.C.) enhanced T cells infiltration. Cluster of differentiation (CD) 4 receptors (CD4^+^) and CD8^+^ infiltrating T cell markers were analyzed by (**A**) immunohistochemical staining and then the number of respective T cells were recorded in each section to determine the percentage of infiltrating T cells from tissues of (**B**) B16F10 and (**C**) LL2 tumor-bearing mice that received either phosphate buffered saline (PBS) or *Salmonella* treatment (×200; Scale bar = 60 μm). (Statistical significance was calculated using Student *t*-test. ** *p* < 0.01, *** *p* < 0.001, compared with PBS, *n* = 3).

Figure 5A shows the tumor size that is apparently different in B16F10 and LL2 tumor-bearing mice. Tumor weight was significantly decreased in the group with *Salmonella* treatment (Figure 5B). Meanwhile, the expression of PD-L1 was lesser in *Salmonella*-treated B16F10 and LL2 tumor-bearing mice as demonstrated by Western blotting (Figure 5C and Figure S5). To analyze the expression of immune cells within the tumor region, tumor tissues were processed for immunohistochemical (IHC) staining using CD4^+^ and CD8^+^ antibodies. *Salmonella* treatment in mice bearing B16F10 and LL2 cells showed a higher number of CD4^+^ and CD8^+^ T cell infiltrations compared with the PBS group (Figure 6A–C). This is consistent with a previous study that also showed significantly higher CD8^+^ T cell infiltrations than CD4^+^ T cells in tumor-bearing mice after treatment with *Salmonella* [8]. These results suggested that systematic administration of *Salmonella* suppressed tumor growth and attracted T cell infiltrations by down-regulating PD-L1.

## 3. Discussion

To date, immunotherapy is considered as a promising strategy for cancer therapy. Targeting the PD-1/PD-L1 axis could reactivate cytotoxic T cells and mount an attack against various solid cancers. However, the treatment effect in patients who received PD-1 and PD-L1 monoclonal antibodies is still limited. Combinational therapeutic approaches led the immune system to attack healthy cells causing severe immune-related adverse events (irAE) in different tissues, such as the skin, gastrointestinal, pancreas, nerve, and endocrine tissues [21]. In some cases, patients with limited or poor response to immunotherapy might be accounted for by the insufficient CD8+ T cell infiltration into non-inflamed types of tumor microenvironment caused by tumor heterogeneity, hypoxia, and specific mutations in oncogene pathways [22,23,24]. Nonetheless, various strategies have already been explored to maximize the potential of targeting PD-1/PD-L1 interaction in combating cancer. A new fusion protein comprising anti-PD-L1 that is genetically fused to tumoricidal protein tumor necrosis factor (TNF)-related apoptosis inducing ligand (TRAIL) bound to PD-L1 expressed on tumor or myeloid effector cells such as monocytes, macrophages, and dendritic cells, causes secretion of IFN-γ by activating T cells. Meanwhile, cells expressing high levels of PD-L1 are sensitized to anti-PD-L1:TRAIL, and this converts them into pro-apoptotic cells [25]. This new fusion anti-PD-L1:TRAIL protein has no negative effects on T cells and might be a potential PD-L1/PD-1 immune checkpoint inhibitor.

Using facultatively anaerobic, attenuated *Salmonella* not only overcame the barriers that traditional therapy cannot break through, but also reduced PD-L1 expression in various murine and human cancer cell lines (Figure 1B and Figure 2B). In another study, when *Salmonella* was treated synergistically with IFN-γ, it reduced the cytokine production through increasing PD-L1 expression in intestinal epithelial cells (IECs) [26]. Nevertheless, in the tumor immunosuppressive microenvironment, the mechanism of *Salmonella* in inhibiting tumor growth involves not only recruiting peripheral natural killer and T cells but also cross-presentation of tumor antigens [27]. On top of these, the present study also showed that immune checkpoint molecules played an important role as a target in the multifaceted interplay between *Salmonella* and tumor cells. Based on our previous studies, *Salmonella* effectively targeted solid tumors, showing marked colonization in livers and spleens [8]. Similar results could be found in different strains of *Salmonella* [10]. In addition to that, *Salmonella* also inhibited angiogenesis in both murine melanoma and breast cancer models, which is another crucial mechanism to incapacitate pro-tumor activities [28]. Even in this study, using the same *Salmonella* wherein we investigated the relationship between *Salmonella*-infected tumor and the immune cells, we are starting to understand how the bacteria targeting tumors alters the tumor microenvironment in a multitude of ways and how these changes at the molecular level could positively affect immune cells to act against the tumor. Different tumor cells have different responses after *Salmonella* treatment. Murine 4T1 and B16F10 tumor cells treated with various M.O.I. *Salmonella* responded in a dose-dependent manner. Meanwhile, human PC9 tumor cells treated with *Salmonella* had a better dose-response than Human H1299 tumor cells. As shown in Figure S1B, the infectious efficiency of *Salmonella* or distribution of receptors, such as TLR4, may influence the signaling transduction after *Salmonella* treatment. 

Although *Salmonella* undoubtedly decreases the expression of PD-L1, it does not affect PD-L2, which might be because it is regulated by Th2 signals, IL-4Rα, and Stat6, whereas PD-L1 is regulated by TLR4-dependent signaling, or mainly restricted to dendritic cells [26,29]. Nevertheless, the role of PD-L2 in cancer progression is still unclear. On another note, phosphoinositide 3-kinase (PI3K) and mitogen-activated protein kinase (MAPK) are crucial oncogenic pathways that regulate PD-L1 expression [30]. Transcriptional PD-L1 is not only regulated by the PI3K pathway [31], but also regulated by signal transducer and activator of transcription 3 (STAT3) [32], nuclear factor kappa B (NF-κB) [33], and B-cell CLL/lymphoma 3 (Bcl3) proto-oncogene [34]. Herein, we illustrated that the P-AKT/P-mTOR/P-70s6K pathway is a prime signaling pathway that is negatively influenced by *Salmonella* treatment in a tumor mice model while the MAPK pathway needs further investigation.

Although *Salmonella* is documented to overcome many of the current barriers to cancer treatment, there is still a long way to go to determine how it can be applied to clinical trials. Currently, VNP20009 and *S.* typhi Ty21a have been recorded to undergo a Phase I human clinical trial. Genetically modifying the gene of VNP20009 purI and msbB attenuated virulence and reduced septic shock, respectively [35]. Carried vascular endothelial growth factor receptor 2 (VEGFR-2) of *S. typhi* Ty21a delivered VXM01, which is an orally available T-cell vaccine used to activate T-cells to breakdown tumor vasculature [36]. *S. typhimurium* A1-R combined with chemotherapy can efficiently target cells in the S/G2/M phases, rather than in the S/G2 phases, hence making it a promising strategy to suppress tumor growth [37]. Utilizing *S. typhimurium* A1-R to assess antitumor efficacy in a patient-derived orthotopic xenograft (PDOX) model may be used as an assessment. Indeed, the use of PDOX models has been documented in human melanoma, pancreatic cancer, osteosarcoma, and gastrointestinal stromal tumor [38]. However, the problems in *Salmonella* need to be further confirmed for clinical feasibility, dose-limiting toxicity, tumor localization, inflammatory reaction, and host immunity [39,40].

## 4. Materials and Methods

### 4.1. Bacteria, Cell Lines, Plasmids and Animal

*Salmonella* enterica serovar Choleraesuis, herein referred to as *Salmonella Choleraesuis* or S.C., (ATCC 15480, Bioresources Collection and Research Center, Hsinchu, Taiwan) was used in this study. Bacteria were maintained in Lysogeny broth (LB) plates and propagated in L.B. broth prior to use. Mouse breast cancer cells (4T1), mouse melanoma cells (B16F10), mouse colon cancer cells (CT26), mouse lung carcinoma (LL2), and human non-small cell lung carcinoma (NSCLC) cells (A549, H1299, and PC9) were maintained in Dulbecco’s Modified Eagle’s Medium (DMEM) supplemented with 100 U/mL penicillin and 100 μg/mL streptomycin, 2 mM l-glutamine and 10% fetal bovine serum at 37 °C. Human non-small cell lung carcinoma cells were kindly provided by Chih-Jen Yang (Department of Internal Medicine, Kaohsiung Municipal Ta-Tung Hospital, Kaohsiung, Taiwan). The EL4 cell line (mouse T lymphocyte; (ATCC TIB-39™)) was a kind gift from Professor Hui-Chen Chen (China Medical University, Taichung, Taiwan) and was maintained in Roswell Park Memorial Institute (RPMI) medium 1640 containing 10% fetal bovine serum (FBS) and 17.3 M 2-mercaptoethanol. The myr-AKT (constitutively active AKT) plasmid was previously described [41]. BABL/c or C57BL/6 mice were purchased from the National Laboratory Animal Center of Taiwan. The Laboratory Animal Care and Use Committee of the National Sun Yat-sen University approved the animal experimental protocol (permit number: 10801).

### 4.2. Western Blot Analysis

The Bicinchoninic Acid (BCA) protein assay (Pierce Biotechnology, Rockford, IL, USA) was used to determine the protein contents. Sodium dodecyl sulfate polyacrylamide gel electrophoresis (SDS-PAGE) was used to fractionate proteins, and the fractionated proteins were transferred to nitrocellulose membranes (Pall Life Science, Glen Cove, NY, USA). The antibodies against PD-L1 (GeneTex, Inc. Irvine, CA, USA), phosphorylation-AKT (Santa Cruz Biotechnology Inc, Santa Cruz, CA, USA), AKT (Santa Cruz), phosphorylation- p70s6K (Cell Signaling, Danvers, MA, USA), p70s6K (Cell Signaling), phosphorylation-mTOR (Cell Signaling), mTOR (Cell Signaling), caspase 3 (GeneTex), or β-actin (Sigma-Aldrich, St. Louis, MO, USA) were used to detect the targeted protein. Rabbit anti-mouse immunoglobulin G (IgG)-peroxidase antibody (Sigma Aldrich) and goat anti-rabbit IgG-peroxidase antibody (Sigma Aldrich) were used as secondary antibodies. A chemiluminescence system (T-Pro Biotechnology, New Taipei City, Taiwan) was used to observe the signals. ImageJ software was used to quantify the signals [41,42].

### 4.3. Cell Viability Assay

4T1 (5 × 10^5^ cells/well), B16F10 and CT26 (3 × 10^5^ cells/well), LL2 and PC9 (7 × 10^5^ cells/well), and H1299 (5 × 10^5^ cells/well) cells were plated in 6-well plates. Each well was treated with the respective dose (M.O.I. = 0, 1, 10, and 100) of Salmonella for 90 minutes and cultured in free DMEM with 1% Gentamicin for 24 hours. Cells were trypsinized, harvested, and stained with tryphan blue. Then, the number of viable cells were counted using a Luna™ automated cell counter (Logos Biosystems, Inc., Gyeonggi-do, South Korea).

### 4.4. Flow Cytometry

B16F10 (1 × 10^5^ cells/well) and LL2 (2 × 10^5^ cells/well) cells were plated in a 6-well plate, infected with 100 M.O.I. After 1.5 h treatment, *Salmonella*, the *Salmonella*-treated cells or PBS-treated cells were cocultured with 10^5^ EL4. EL4 cells were collected after 24 hours. For PD-L1 detection, 10^6^ cells were counted and fixed with 70% ethanol in −30 °C overnight. Subsequently, primary antibody was added and let to stand for 1 h at 4 °C and then with fluorochrome-labeled goat anti-rabbit IgG secondary antibody (GeneTex) for another 30 min at 4 °C. To analyze the cell cycle, cells were fixed with 70% ethanol in −30 °C overnight. Cells were reacted with 5 μg/mL propidium iodide (PI, GeneTex) and 10 mg/mL RNase A (Allbio Life Co., Ltd, Taichung, Taiwan) for 30 min at room temperature. To detect apoptotic cells, cells were treated with AnnexinV-Fluorescein isothiocyanate (FITC) Apoptosis Detection Kit (Strong Biotech Co., Taipei, Taiwan) according to the manufacturer’s instructions. The fluorescence of 10,000 cells per sample was measured and analyzed using Attune NxT Flow Cytometer (Life Technologies, Carlsbad, CA, USA).

### 4.5. Cytokine Measurement

B16F10 and LL2 cells were treated with 100 M.O.I. *Salmonella* and co-cultured with mouse splenocytes (3 × 10^5^–3 × 10^6^ cells/well). Supernatants were collected and the IL-12 and IFN-γ were measured using a BD OptEIA kit (BD Diagnostic, San Diego, CA, USA) according to the manufacturer’s instructions after 24 h co-culture.

### 4.6. Animal Studies

C57BL/6 mice were inoculated subcutaneously (s.c.) with 106 B16F10 and LL2 cells. The tumor-bearing mice were intraperitoneally (i.p.) injected with PBS or *Salmonella* 2 × 10^6^ colony-forming units (cfu) on day 7. Groups of B16F10 and LL2 tumor-bearing mice were measured for tumor weight and immunoblotting assay at day 15 and 21, respectively.

### 4.7. Immunohistochemical Staining

C57BL/6 mice were inoculated subcutaneously with 106 B16F10 and LL2 cells, respectively. The mice were treated with PBS or *Salmonella* 2 × 10^6^ cfu on day 12. The tumor cells were removed, weighed, washed with PBS, fixed in 3.7% formaldehyde, and embedded in paraffin after 2 days of infection with *Salmonella*. Tissues were processed in 5-μm sections and stained with rat anti-mouse CD4 (GeneTex, Inc.), or mouse anti-mouse CD8 (GeneTex, Inc.) antibody. After sequential incubation with appropriate peroxidase-labeled secondary antibody and 3′,3′-diaminobenzendine (DAB) as substrate chromogen, the slides were counterstained with hematoxylin.

### 4.8. Statistical Analysis

The experimental data were analyzed and presented as mean ± standard deviation (SD). Student’s *t*-test was used to assess statistically significant differences. A *p*-value less than 0.05 was considered to be statistically significant.

## 5. Conclusions

Concerted changes in pro-tumor activities in the tumor microenvironment rely not only on tumor-intrinsic cellular processes but also on tapping into the activities of infiltrating immune cells, which then promote tumor cell survival, proliferation, and progression. We previously reported that *Salmonella,* as an antitumor agent, reverses these changes. In brief, our present findings revealed that the tumor-induced immunosuppressive molecule, PD-L1, was downregulated by *Salmonella* via the AKT/mTOR/p70s6K pathway, stimulated Th1 related cytokine secretion, and reactivated effector T cells, achieving significant tumor suppression.

## Supplementary Materials

The following are available online at https://www.mdpi.com/2072-6694/12/1/57/s1, Figure S1: (A) Quantified band intensities of respective PD-L1 proteins in various cancer cell lines. (B) Quantified PD-L1 proteins in Salmonella-infected cell lines. Statistical significance was calculated using Student t-test. *, p < 0.05; **, p < 0.01, Figure S2: (A) Quantified band intensities of respective proteins in Salmonella-infected cell lines. (B) PD-L1 expression of Salmonella-infected cell lines as assessed by flow cytometry analysis. Statistical significance was calculated using Student t-test. *, p < 0.05; **, p < 0.01; ***, p < 0.001, Figure S3: (A) Quantified band intensities of cleavage caspase 3 (17/19 kDa) proteins in EL4 co-cultured with cells, compared with each PBS. The inserted values indicate protein expression compared to β-actin. Mean ± SD represented independent expression. Statistical significance was calculated using Student t-test. **, p < 0.01, Figure S4: (A) Quantified band intensities of respective proteins of tumor cells bearing constitutively active-AKT. (B) Quantified band intensities of caspase 3 (17/19 kDa) protein of EL4 cocultured with tumor having constitutively active-AKT. The inserted values indicate protein expression compared to β-actin. Mean ± SD represented independent expression. Statistical significance was calculated using Student t-test. *, p < 0.05; **, p < 0.01; ***, p < 0.001, Figure S5: Quantified band intensities of tumor-bearing mice PD-L1 protein, Figure S6: (A) Western blotting of cell lysates harvested from different cancer cell lines under normal culture condition revealed varying levels of PD-L1. (B) Western blotting of Salmonella dose-dependently inhibited PD-L1 expression, Figure S7: Western blotting of respective proteins in Salmonella-infected cell lines, Figure S8: (A) Western blotting of respective proteins of tumor cells bearing constitutively active-AKT. (B) Western blotting of caspase 3 protein of EL4 co-cultured with tumor having constitutively active-AKT.
