# Supplementary material for: Salmonella Breaks Tumor Immune Tolerance by Downregulating Tumor Programmed Death-Ligand 1 Expression
Source: Cancers (Basel). 2019 Dec 24;12(1):57. doi: 10.3390/cancers12010057 (PMC7017279; doi:10.3390/cancers12010057)
Supplement: Supplementary file 1 [file cancers-12-00057-s001.pdf]

Supplementary Materials

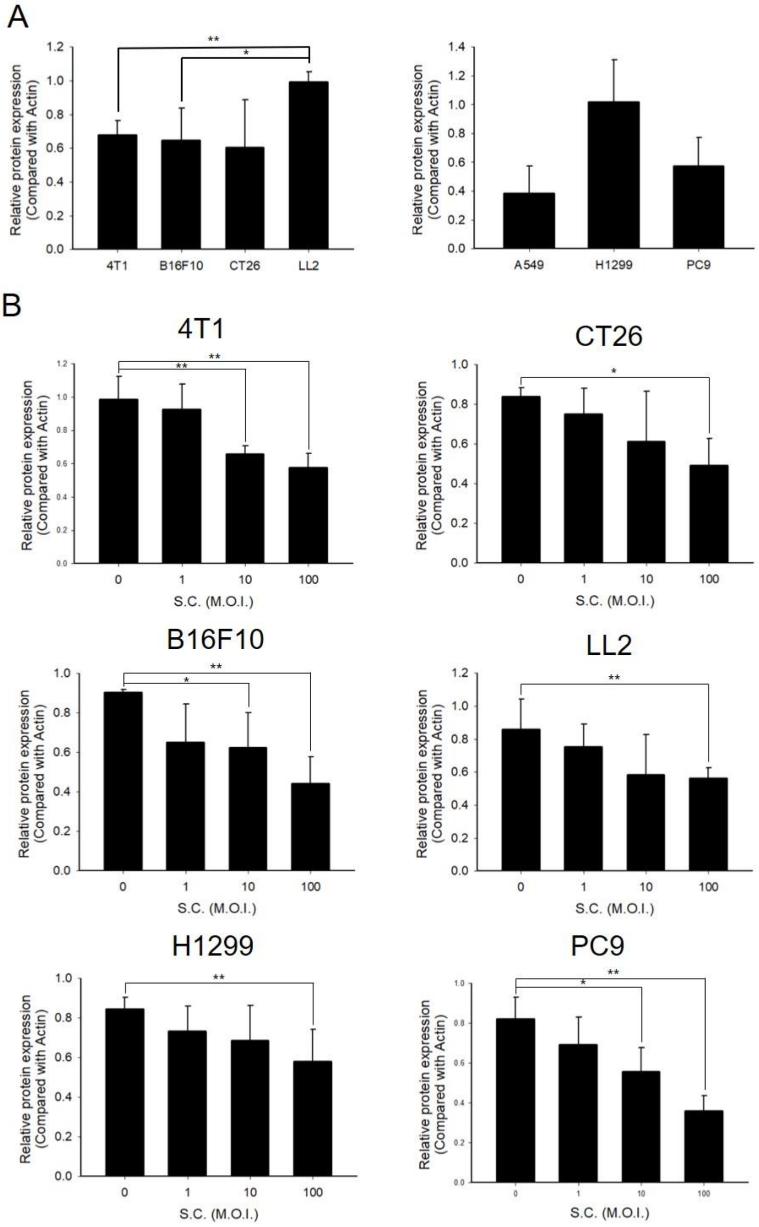

Figure S1. (**A**) Quantified band intensities of respective PD-L1 proteins in various cancer cell lines. (**B**) Quantified PD-L1 proteins in *Salmonella*-infected cell lines. Statistical significance was calculated using Student t-test. *, $p < 0.05$; **, $p < 0.01$.

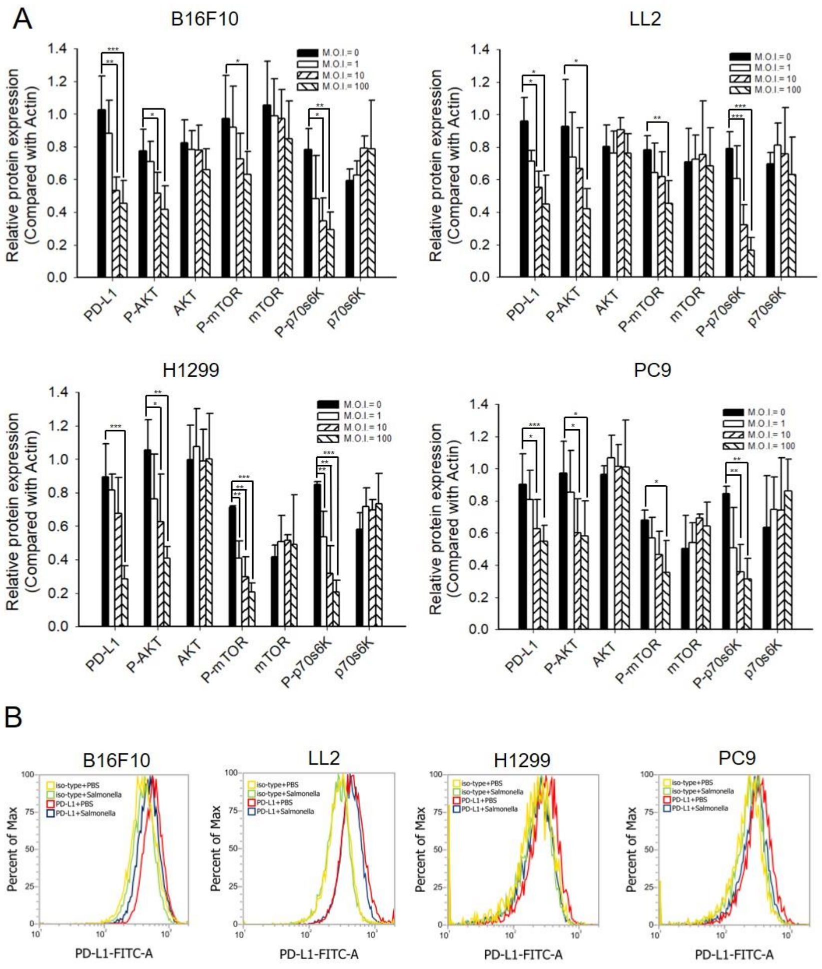

Figure S2. (**A**) Quantified band intensities of respective proteins in *Salmonella*-infected cell lines. (**B**) PD-L1 expression of *Salmonella*-infected cell lines as assessed by flow cytometry analysis. Statistical significance was calculated using Student t-test. *, $p < 0.05$; **, $p < 0.01$; ***, $p < 0.001$.

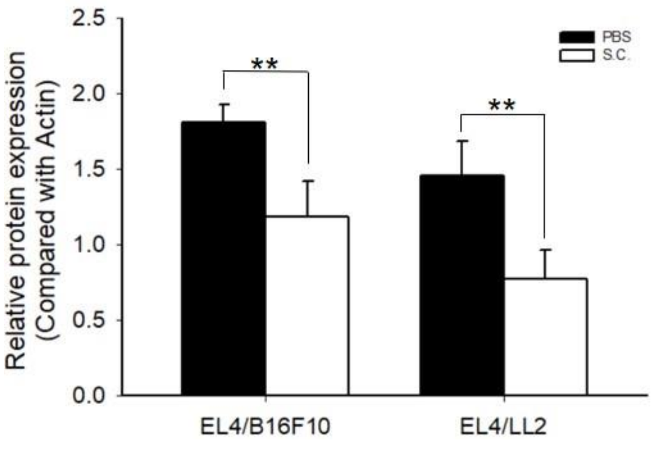

Figure S3. (**A**) Quantified band intensities of cleavage caspase 3 (17/19 kDa) proteins in EL4 co-cultured with cells, compared with each PBS. The inserted values indicate protein expression compared to β-actin. Mean ± SD represented independent expression. Statistical significance was calculated using Student t-test. **, $p < 0.01$.

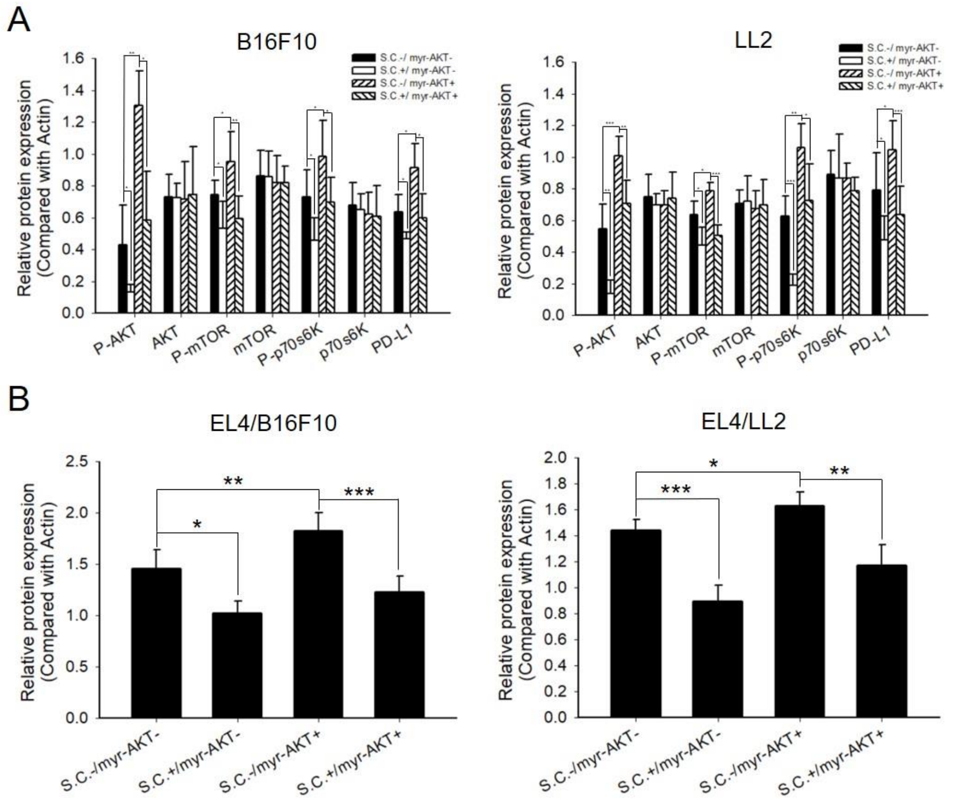

Figure S4. (**A**) Quantified band intensities of respective proteins of tumor cells bearing constitutively active-AKT. (**B**) Quantified band intensities of caspase 3 (17/19 kDa) protein of EL4 cocultured with tumor having constitutively active-AKT. The inserted values indicate protein expression compared to β-actin. Mean ± SD represented independent expression. Statistical significance was calculated using Student t-test. *, $p < 0.05$; **, $p < 0.01$; ***, $p < 0.001$.

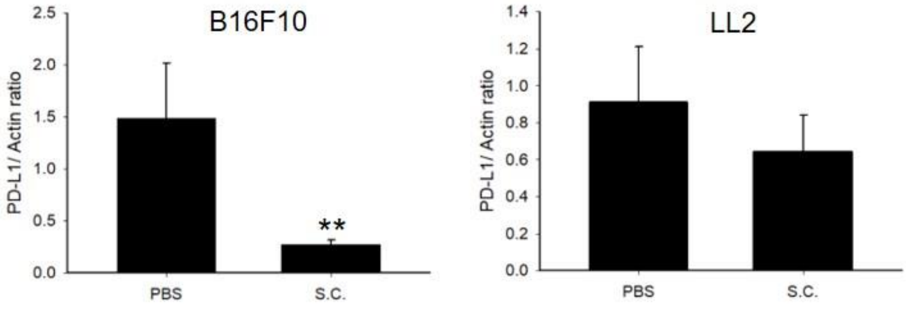

Figure S5. Quantified band intensities of tumor-bearing mice PD-L1 protein. The inserted values indicate protein expression compared to β-actin. Mean ± SD represented three to four times independent expression. Statistical significance was calculated using Student t-test. **, $p < 0.01$, compared with PBS.

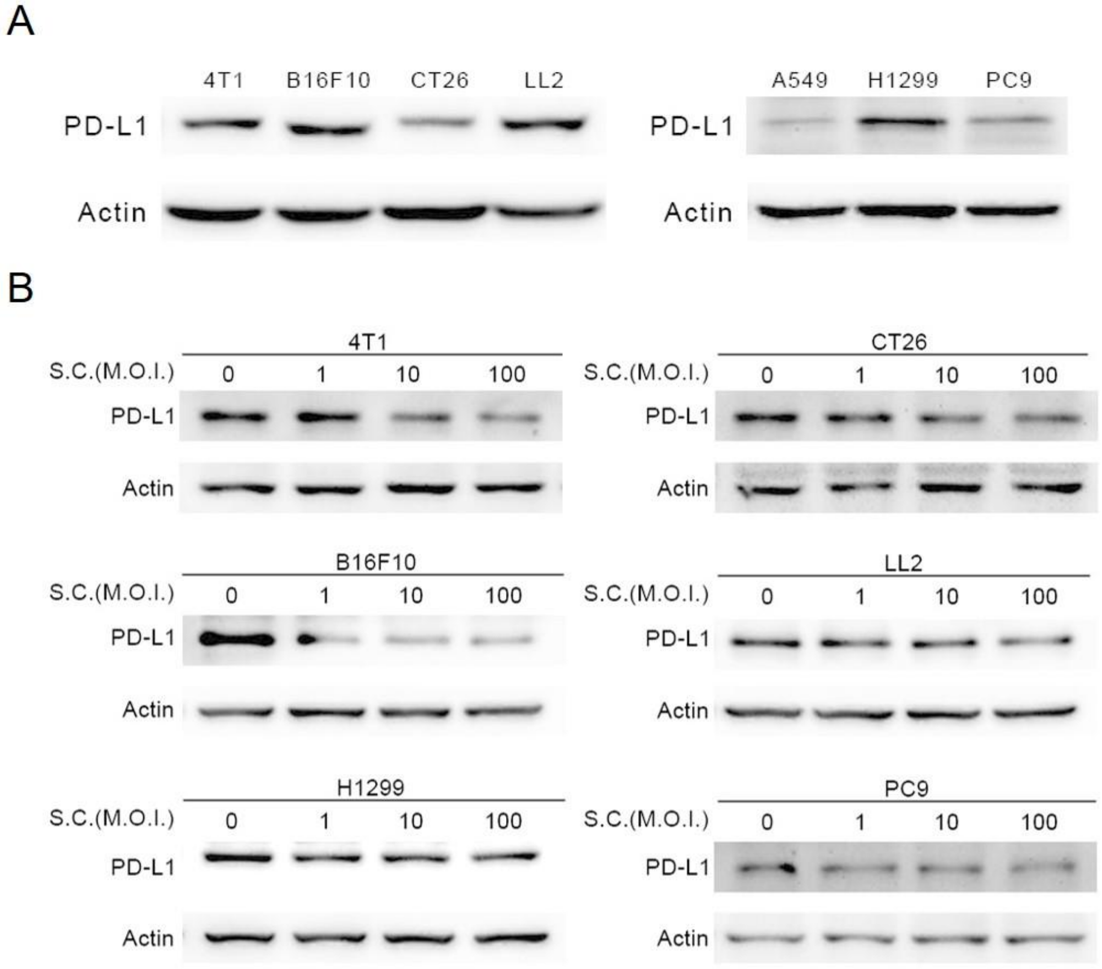

Figure S6. (**A**) Western blotting of cell lysates harvested from different cancer cell lines under normal culture condition revealed varying levels of PD-L1. (**B**) Western blotting of *Salmonella* dose-dependently inhibited PD-L1 expression.

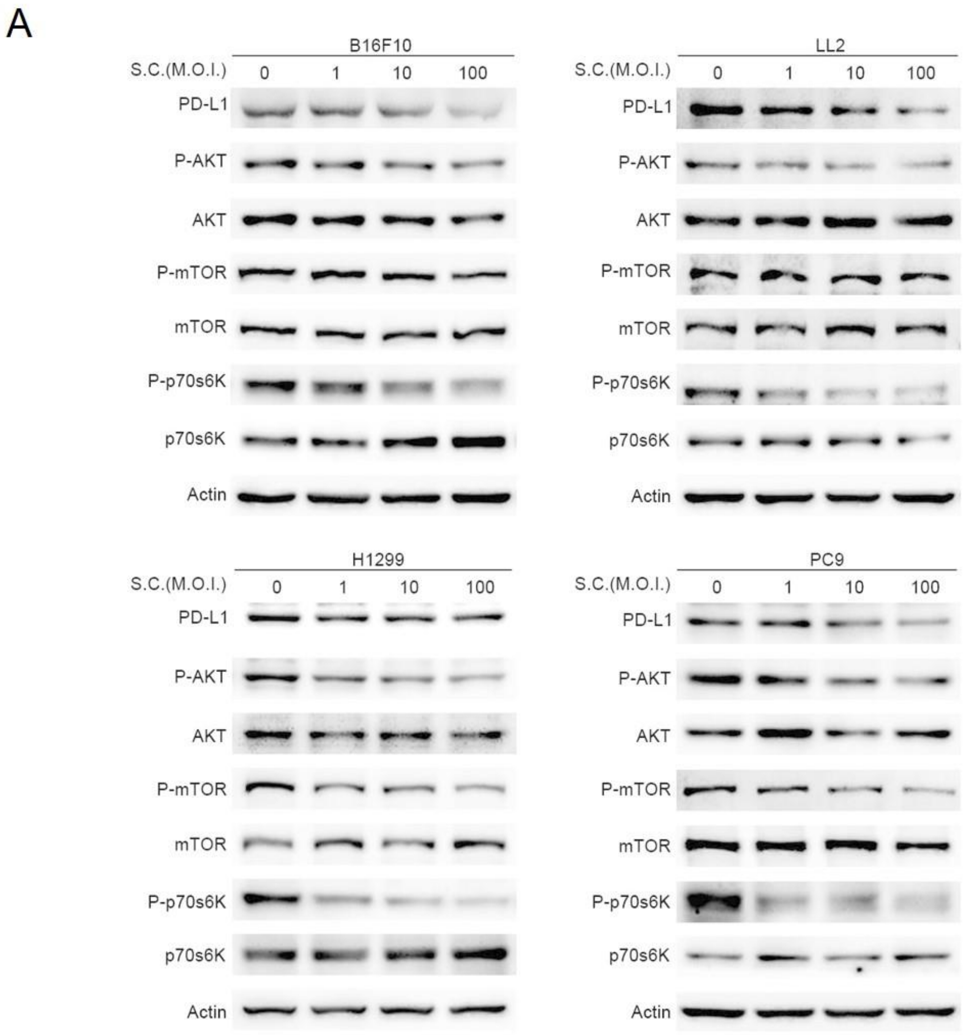

Figure S7. Western blotting of respective proteins in *Salmonella*-infected cell lines.

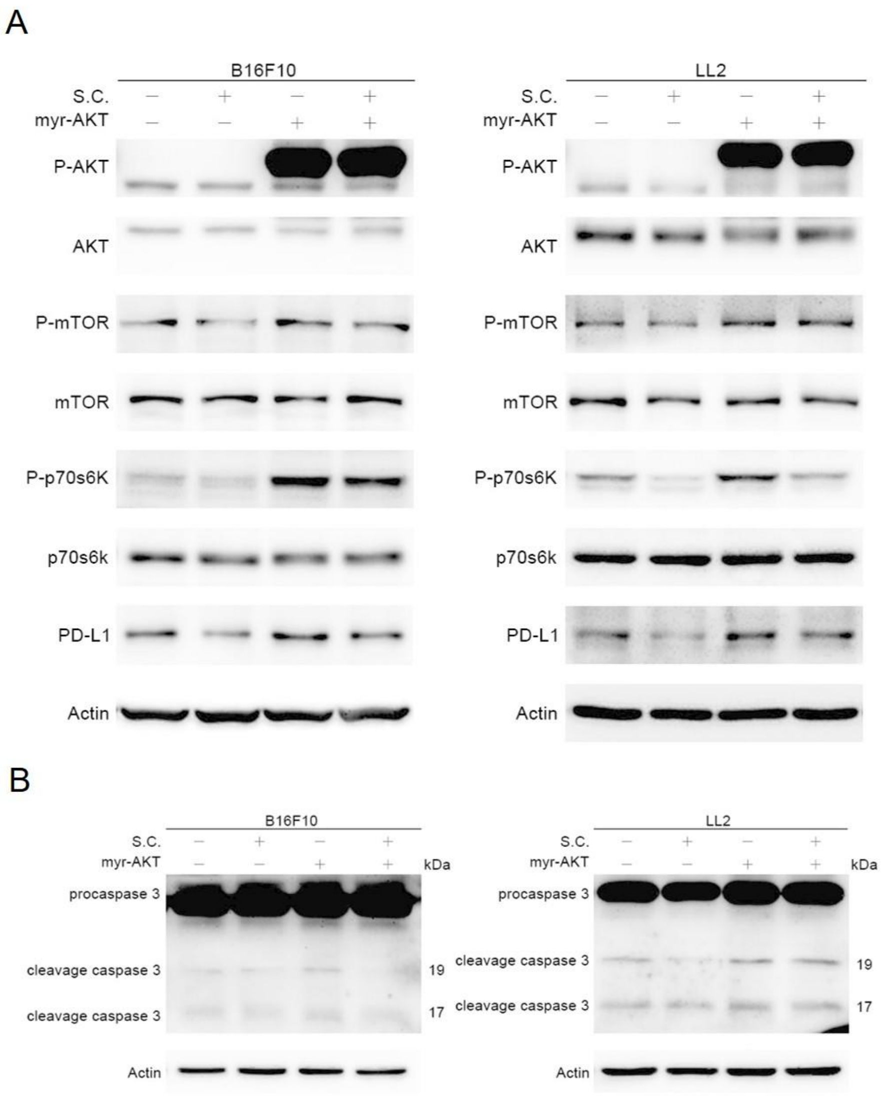

Figure S8. (**A**) Western blotting of respective proteins of tumor cells bearing constitutively active-AKT. (**B**) Western blotting of caspase 3 protein of EL4 co-cultured with tumor having constitutively active-AKT.
